# Aging Profiles of AlSi7Mg0.6 and AlSi10Mg0.3 Alloys Manufactured via Laser-Powder Bed Fusion: Direct Aging versus T6

**DOI:** 10.3390/ma15176126

**Published:** 2022-09-03

**Authors:** Emanuela Cerri, Emanuele Ghio

**Affiliations:** Department of Engineering and Architecture, University of Parma, Via G. Usberti, 181/A, 43124 Parma, Italy

**Keywords:** AlSi10Mg and AlSi7Mg alloys, aging profiles, direct aging heat-treatments, T6 heat-treatment, heat-treatment optimization, Laser Powder Bed Fusion

## Abstract

The artificial aging heat treatments performed directly on as-built and solubilized AlSi7Mg0.6 and AlSi10Mg0.3 samples were characterized and discussed. The analysed bars and billets (height of 300 mm) were manufactured via the Laser Powder-Bed Fusion process on a build platform heated at 150 °C. Therefore, its influence on the as-built samples was studied in terms of mechanical performance variations between the bottom and top regions. Vickers microhardness measurements were performed to obtain aging profiles after direct aging (175–225 °C) and T6 heat treatments and to highlight better time and temperature parameters to optimize the mechanical properties of both alloys. SEM observations were used to characterize the microstructure before and after the heat treatments and its influence on the fracture mechanisms. Generally, the direct aging heat treatments show the same effects on both aluminium alloys, unlike the solubilization at 505 °C followed by artificial aging at 175 °C. The strengths vs. elongation values obtained after the direct aging treatments are better than those exhibited by T6 as highlighted by the quality index.

## 1. Introduction

Laser Powder-Bed Fusion (LPBF) is an Additive Manufacturing (AM) technology for metals and alloys, where one or more lasers scan a powder bed (layer) on a cold or hot build platform. This layer-by-layer process aims to manufacture geometrically complex components which can be also characterized by high customization. LPBF also minimizes the material waste and reduces the costs and time of production, namely the advantages conferred by the AM processes. At the same time, the complex software allows obtaining a weight reduction of the designed mechanical or structural components thanks to careful optimization of their geometry. In fact, the metal material will be distributed within specified design space in accordance with the Finite Element Analysis (FEA) results [1,2,3]. For these reasons, the LPBF process is good for several industrial applications, e.g., automotive and aerospace thanks to the possible production of commercial and military vehicles and/or aircraft structures [4,5,6].

In this scenario, the hypo-eutectic AlSi7Mg0.6 and AlSi10Mg0.3 alloys find numerous uses owing to their high specific strength conferred by their low density (ρ=2.68 g/cm^3^) and to the good corrosion resistance due to the oxide film [7,8]. Both the cast A357 (AlSi7Mg0.6) and AlSi10Mg0.3 aluminium alloys belong to the age-hardening Al-Si-Mg ternary system where the 0.3 and 0.6% of Mg promote the following precipitation phenomena [9]: 

SSS → GP zone → β″ coherent precipitate → β′ semi-coherent precipitate → β-Mg_2_Si incoherent precipitate

Therefore, the age hardening response promotes firstly the formation of the GP (Guinier–Preston) zones, which are extremely fine-scaled solute enriched regions, from the SSS and, secondly, their evolution in coherent β″ (Mg_5_Si or Mg_5_Al_2_Si_4_), semi-coherent β′ (Al_3_Mg_9_Si_7_) and incoherent β-Mg_2_Si precipitates [9,10,11,12]. Focusing on Al-Si-Mg alloys manufactured via the LPBF process, the high cooling rate (~10^6^ K/s) causes an initial Si concentration in α-Al matrix higher than the equilibrium content of 1.65% [13,14]. Therefore, the mechanical performance of both aluminium alloys can be improved through both T5 and T6 heat treatments (HTs). In the former case, the ultimate tensile strength (UTS), yield strength (YS), and hardness are improved by performing artificial ageing (AA) at about 160 °C. In the latter case, the AA follows the solubilization heat treatment (SHT) at about 500–540 °C for different holding time, and makes it possible to obtain higher UTS, YS and hardness than the T5 as discussed by [15]. Perreira et al. [16] reported (321 ± 2) MPa and (271 ± 2) MPa of UTS and YS, respectively, and (118 ± 12) HV after a standard T6 (535 °C × 0.25 h + 160 °C × 10 h) performed on cast AlSi7Mg0.6. Van Cauwenbergh et al. [14] showed, however, (228 ± 16) MPa for UTS and (183 ± 33) MPa for YS of a cast AlSi10Mg0.3 alloy heat treated at (540 °C × 8 h + 160 °C × 6 h). The elongation reaches (1.50 ± 0.40) %, a very low value as for the previously mentioned A357-T6 showing (2.9 ± 0.3) %. The same mechanical properties were reported in [14,16]. Kempen et al. [17] reported an HV improvement form 95–105 HV in as-cast conditions to 130–133 HV after the T6 on AlSi10Mg0.3 cast alloy. 

Focusing on the as-built AlSi7Mg0.6 and AlSi10Mg0.3 alloys, the conventional T6 HT generally confers detrimental effects for the UTS, YS and hardness values. As a matter of fact, the full-cellular structure where the α-Al matrix is surrounded by nano-size Si-eutectic particles distribution is completely destroyed by the high temperatures reached during the SHT [18,19]. From a three-dimensional point of view, the eutectic particles formed a tubular structure arranged along with the build direction (i.e., parallel to the build platform) which confers UTS and YS higher than 360 MPa and 210 MPa, respectively, for the as-built AlSi10Mg0.3 alloy [9]. Comparable mechanical properties are shown by the as-built AlSi7Mg0.6 alloy [20,21]. As widely discussed in [9,22], the pre-heated (hot) build platform plays fundamental roles on both the precipitation phenomena and the residual stress amount and, consequently, on the obtained mechanical performance. At the same time, the SSS formed thanks to the high cooling rate characterizing the LPBF process fails. As regards the ductility, the as-built values (<9%) do not satisfy the current standard specifications yet. The microstructural changes induced by the T6 HT lead the UTS and YS at values lower than 300 MPa and 250 MPa, respectively, without a satisfactory improvement of the elongation values. In addition, the high temperatures reached during the SHT induces a pore size increment as widely reported by [23,24] and discussed in [25] for the AlSi10Mg0.3 alloy. Tonelli et al. [26] added that the T6 HT can reduce the microstructural anisotropy and the correlated mechanical properties, in addition to the completely relieve of the residual stress after 10′ at 540 °C in AlSi7Mg0.6 subjected to LPBF. To reduce the residual stress and nullify the sample distortions when it is separated from the build platform, the stress relief at 300 °C × 2 h is suggested instead of the T6 [9]. In fact, the former confers better mechanical performance than the latter. 

In relation to the build platform temperature and the total height of the manufactured samples, direct aging (DA) HTs at temperatures lower than 250 °C can lead to greater optimized mechanical properties, also through a reduction of the processing time with respect to T6 HT. As a matter of fact, the SSS of the as-built AlSi7Mg0.6 and AlSi10Mg0.3 samples can trigger the precipitation phenomena already during the DA [11]. In addition, the full-cellular structure continues to preserve the mechanical properties of the heat-treated samples [9]. 

The present work aims to evaluate the effects induced by T6 HT on the AlSi7Mg0.6 subjected to LBPF in terms of microstructure, porosity and mechanical performance, and to compare them to the effects obtained by the L-PBFed AlSi10Mg0.3 alloy in the same heat-treated conditions. In addition, different time of SHT were also considered and analysed. At the same time, it presents the best impact conferred on the same aluminium alloys by different direct aging heat-treatments also considering the effects induced by the pre-heated build platform. 

## 2. Materials and Methods

The set of powders used to manufacture the samples analysed in the present work are AlSi7Mg0.6 and AlSi10Mg0.3. Table 1 listed their nominal chemical compositions. In both cases, the particles diameter had a distribution of 20–63 μm and the apparent density is higher than 1.52 g/cm^3^.

The LPBF process was conducted on an SLM^®^280 machine where two IPG fibre lasers, characterized by a maximum power of 700 W, work in parallel manufacturing samples both in single-laser (SL) and double-laser (DL) zones, respectively. In the former zone, only one laser scans the powder bed; in the latter one, the powder bed is scanned simultaneously by two lasers. The design of the printed samples, where the yellow and red areas of the build platform represent the SL and DL zones, is shown in [25]. For both aluminium alloys, bars (10 × 10 × 300 mm^3^) and billets (10 × 100 × 300 mm^3^) were manufactured with the same process parameters (Table 2) but in different processing times. AlSi10Mg0.3 billets and bars are manufactured in 54 h, while AlSi7Mg0.6 samples were printed in 75 h. This difference in manufacturing time was dictated by a slowdown in the powder bed deposition.

**Figure 1:**
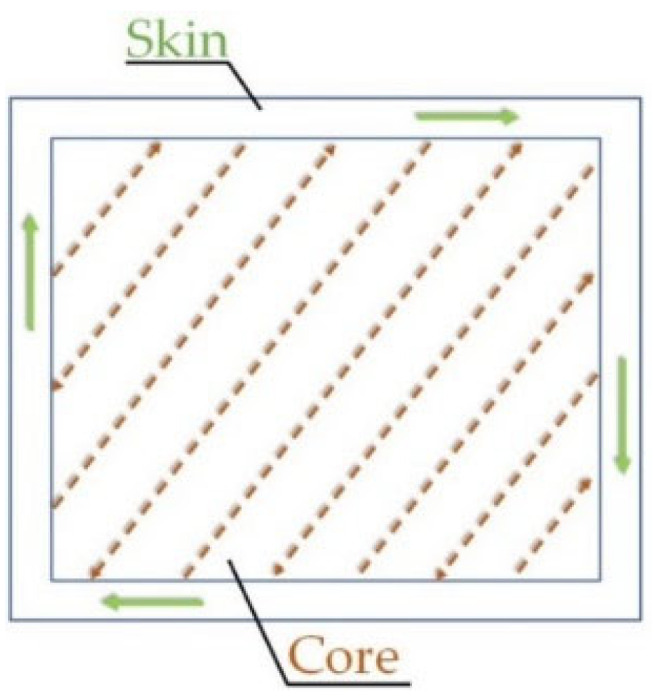
Skin-Core scan strategy used to manufacture AlSi7Mg0.6 and AlSi10Mg0.3 bars and billets. The skin thickness is 200 ± 10 μm.

For a better understanding of the discussed results, all billets and bars are divided into three different groups in relation to their total height: bottom (0–100 mm), middle (100–200 mm) and top (200–300 mm). All samples were manufactured using the skin-score scan strategy represented in Figure 1. The dotted arrows, which indicate the laser scan tracks, are inclined of 56.5° with respect to the skin base. For each deposited layers, the scanning direction is rotated of 67° with respect to the previous scanned and solidified layer. 

The aim of the present study is to analyse the effects induced by the DA and T6 HTs and to identify the optimal conditions on both AlSi7Mg0.6 and AlSi10Mg0.3 alloys. Table 3 summarizes the studied HT conditions and the samples on which the HTs were performed. T6* and T6** will represent the peak-aging conditions for the AlSi10Mg0.3 and AlSi7Mg0.6 alloys, respectively. The temperatures chosen for the DA, SHT and AA heat treatments were based on [18,22], where AlSi10Mg0.3 alloys manufactured with a layer thickness of 50 μm were analysed. The top and bottom samples listed in Table 3 were cut by the bars (see Figure 1 in [25]) and the different heights were chosen in relation to the both the HV measurements performed on the same bars in as-built conditions and to the results obtained in [18,22]. The T5 (i.e., DA) and T6 HTs were carried out following the conditions dictated by the standard specification of aluminium alloy casting [27].

**Figure 2:**
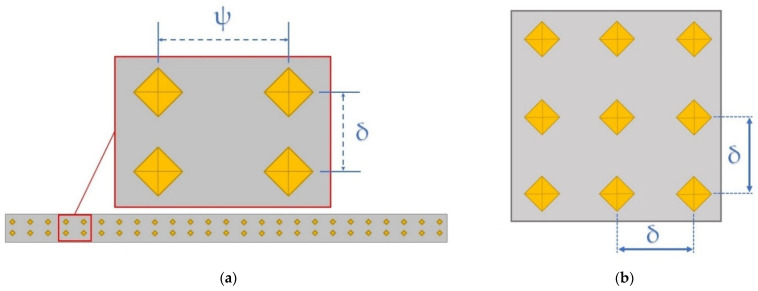
Schematical representation of the HV profiles (**a**) and 3 × 3 matrix (**b**) performed on the xz and xy planes of the bars, respectively. The build directions are: (**a**) parallel to the longer side of the bar, (**b**) perpendicular to the xy plane (i.e., on which the HV is measured).

The HV values were measured with a VMHT Leica (Leica, Wetzlar, Germany) microhardness tester using 500 gf of load and 15 s of indentation time in accordance with UNI EN ISO 6507-1:2018 standard specification. All indentations were performed on the mechanically ground and polished surface of the bars. For the as-built SL and DL bars, the HV measurements were performed on the xz (perpendicular to the build platform) and xy (parallel to the build platform) planes where two HV profiles and a 3 × 3 matrix were built, respectively. Each HV profile was formed by 60 measurements where the distance between two successive indentations was 5 mm (ψ in Figure 2a). A distance of 1 mm was also included to separate the two profiles to avoid the work hardening effect. For each couple of indentations at the same height, the 60 average values of HV were used to plot the HV profiles for the SL and DL bars. Focusing on xy planes, the 3 × 3 matrices were always carried out on the centre of the bar cross-section to avoid the contribution conferred by the skin part (Figure 1) as shown in [25]. The 9 indentations were spaced 1 mm from each other as highlighted by the δ in Figure 2b. The same 3 × 3 matrices were also used to evaluate the microhardness on xy planes of the direct aged solution and T6 heat-treated samples (Table 3). All points forming the aging curves and the SHT profiles, and related to each HT conditions, were calculated as the average of the 9 indentations. The correlated standard deviations were used as error bands. 

The as-built and heat-treated microstructure was analysed by the DMi8 optical microscope (OM) (Leica, Wetzlar, Germany) equipped with LAS-X software through which the image analysis of both the Si-eutectic particles in T6 heat-treated samples and pores was performed. Firstly, one OM micrograph at 500× and three at 1000× of magnification were considered to analyse the Si-eutectic on solution heat-treated samples at 1 h (SHT 1 h), 2 h (SHT 2 h) and 4 h (SHT 4 h). Secondly, the pores analysis was carried out following the methodology used in [25] because the density values of the heat-treated AlSi7Mg0.6 samples will be compared to those discussed for the AlSi10Mg0.3 samples in the same research article. For this reason, the chosen HTs are: DA at 175 °C, 200 °C and 225 °C × 8 h, and T6*. In addition, in this case, a density of 2.68 g/cm^3^ is considered for an AlSi7Mg0.6 sample having 0% in vol. of pores, and all studied micrographs were taken on the xy plane. In summary, six different OM micrographs (magnification of 100×) were systematically analysed to evaluate the relative density of the as-built and heat-treated AlSi7Mg0.6 samples. The micrographs were manually analysed through the LAS-X 2D software to determine the relative density $\left(\rho_{r}\right)$ of each sample. Then, the following equation was considered:

(1) $$\;\rho_{r}=1-\frac{\sum_{i}{\left(A_{p}\right)}_{i}}{\sum_{j=1}^{6}{\left(\hat{A}\right)}_{j}}$$

where $A_{p}$ is the area of a single analyzed pore and $\left[\overset{6}{\underset{j=1}{\sum }}{\left(\hat{A}\right)}_{j}\right]$ is the total area of the considered six OM micrographs. Its value is 24,936.41 × 10^3^ μm^2^. The errors associated to the density values were calculated, as expressed and validated in the Appendix A of the [25], as follows:

(2) $$e_{a}=\frac{\rho_{r}^{max}-\rho_{r}^{min}}{2}=\frac{\left[1-\frac{{\left[\sum_{i}{\left(A_{p}\right)}_{i}\right]}_{min}\;}{\sum_{j=1}^{6}{\left(\hat{A}\right)}_{j}}\right]-\left[1-\frac{{\left[\sum_{i}{\left(A_{p}\right)}_{i}\right]}_{max}\;}{\sum_{j=1}^{6}{\left(\hat{A}\right)}_{j}}\right]}{2}$$

where ${\left[\underset{i}{\sum }{\left(A_{p}\right)}_{i}\right]}_{min}$ and ${\left[\underset{i}{\sum }{\left(A_{p}\right)}_{i}\right]}_{max}$ are the underestimation and overestimation of the total pore area, respectively. Both contributions were obtained by combining the standard errors, listed in Table 4, with each value of the pore area. In detail, these standard errors consider both the errors committed by the operator during the image analysis and the error related to the OM (see [25]). 

**Figure 3:**
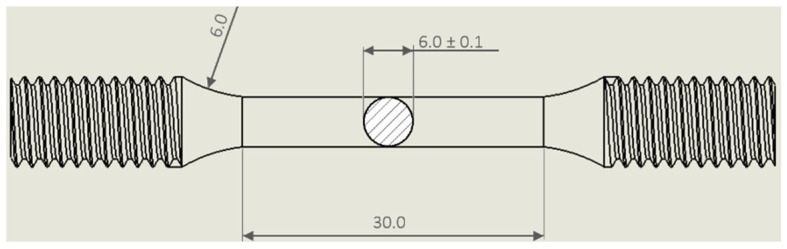
Graphical representation of a cylindrical tensile sample obtained from the as-built billets, where all measurements are expressed in mm (Reprinted from reference [25]).

Tensile tests were conducted at room temperature using the Z100 Zwick/Roell (ZwickRoell, Einsingen, Germany) servo-hydraulic machine with a cross-head speed of 0.008 s^−1^. The cylindrical tensile samples were mechanically obtained by the as-built billets, and their dimensions are illustrated in Figure 3. After the turning process, the tensile samples were heat-treated following the optimal conditions highlighted by the HV measurements to complete them with the tensile properties. Thanks to the machining process, the skin part (Figure 1) was removed and, therefore, its possible detrimental effects on the mechanical performance were nullified, as widely demonstrated in [25]. 

## 3. Results

### 3.1. Density Analysis

**Figure 4:**
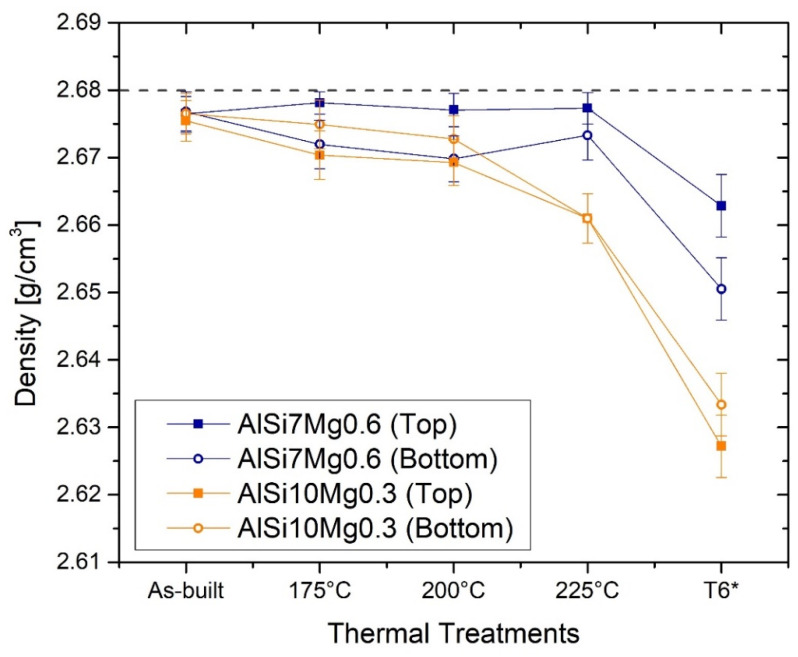
Trends of the density values related to top and bottom of the AlSi7Mg0.6 and AlSi10Mg0.3 samples before and after the direct aging at 175, 200, 225 °C for 8 h and the T6*. The values related to the AlSi10Mg0.3 samples were presented and discussed in [18].

The as-built AlSi7Mg0.6 samples are characterized by a density of 2.667 ± 0.003 g/cm^3^, both in the bottom and top regions. The same density values characterize the as-built AlSi10Mg0.3 samples that show 2.675 ± 0.003 g/cm^3^ and 2.676 ± 0.003 g/cm^3^ for the top and bottom, respectively (Figure 4). Even after the DA at 175 °C × 8 h and 200 °C × 8 h, the density values of the AlSi7Mg0.6 and AlSi10Mg0.3 at the top and bottom regions remain almost unchanged. The first step related to the decrease in density (up to 2.660 ± 0.004 g/cm^3^) is shown by AlSi10Mg0.3 samples after the DA at 225 °C × 8 h; the second is after the T6*, where the density values reach 2.627 ± 0.005 g/cm^3^ and 2.633 ± 0.005 g/cm^3^ for the top and bottom samples, respectively. These latter values are consistent with those exhibited in [18,19,22]. 

**Figure 5:**
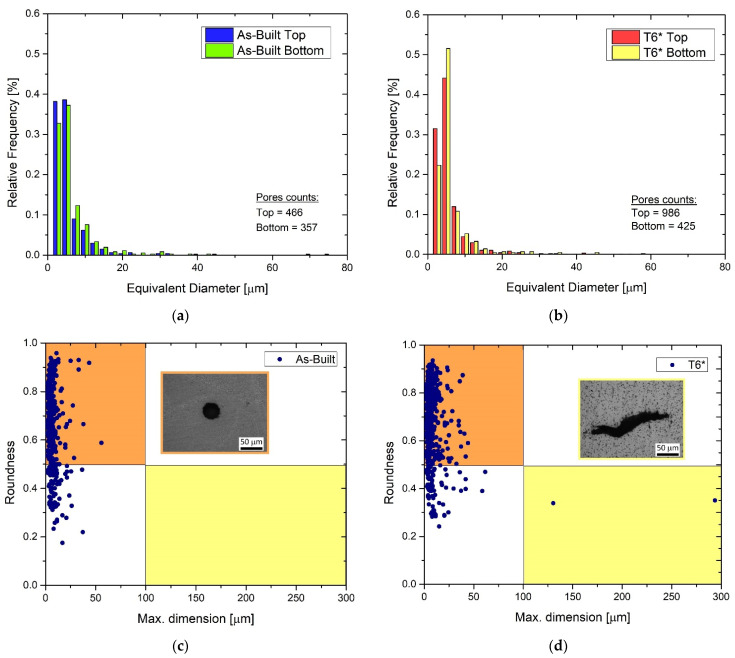
(**a**,**b**) Statistical distribution of the equivalent diameter of the pores and (**c**,**d**) their roundness versus their maximum dimensions in: as-built (**a**,**c**) and heat-treated T6* samples (**b**,**d**).

Focusing on the top and bottom AlSi7Mg0.6 samples in as-built conditions and after the T6*, Figure 5a,b illustrates the relative frequencies of the equivalent diameters of all analysed pores which are classified through the graphs drawn in Figure 5c,d. In this scenario, the as-built top and bottom samples (Figure 5a) approximatively show the same number of pores, 466 for the top and 357 for the bottom regions, characterized by the same equivalent diameters. In detail, the top samples have 38% and 39% of pores with an equivalent diameter of 2.5 and 5 μm, respectively; the bottom samples show 33% and 37%, respectively (Figure 5a). The frequencies decrease to 9% and 12% for the porosity having an equivalent diameter of 7.5 μm on top and bottom regions, respectively. The trend continues to decrease from the last-mentioned values down to zero for equivalent diameters of about 35 μm. Regarding the AlSi7Mg0.6 after the T6*, firstly, the total counted pores increase from the as-built case; secondly, the maximum relative frequencies (44% and 52% for top and bottom samples, respectively) are concentrated on the equivalent diameter of 5 μm (Figure 5b) and are not also distributed on lower values as in Figure 5a. The relative frequency trends strongly decrease to 7.5 μm (Figure 5b), and then decrease up to 0% at around 40 μm. The relative density values of the top and bottom samples are 99.37% and 98.92%, respectively, i.e., 2.663 ± 0.005 g/cm^3^ and 2.651 ± 0.005 g/cm^3^ (see Figure 4). These lower density values of the bottom samples are dictated by the presence of a major quantity of larger pores than in the top samples (>7.5 μm in Figure 5a, >10 μm in Figure 5b). 

Figure 5c,d shows the maximum dimension of the analysed pores, which is the greatest Feret diameter [25], and their roundness (*R*) that is defined as follows [28]:

(3) $$R=\frac{4\pi A}{p^{2}},$$

where *A* and *p* are the area and perimeter of the object (pore in this context) analysed through the image analysis. This object/pore tends to be a circle if *R*→1, while it becomes less round when *R*→0. At the same time, the orange and yellow areas group together the metallurgical (<100 μm) and lack-of-fusion (>100 μm) pores which are illustrated in the OM micrographs in Figure 5c,d, respectively. From these premises, all analysed pores in as-built samples (Figure 5c) can be considered as metallurgical pores and, therefore, formed by the gas dissolved within the molten pools. After the T6* HT, the increase in pore quantity is also highlighted, as well as their tendency to hire a more circular shape (Figure 5d). In fact, the point distribution is shifted upwards. In addition, two LOF pores were found within the analysed area. Considering their formation mechanisms, both of LOF pores are certainly generated during the L-PBF process. The SHT can only promote spheroidization phenomena [9,22].

### 3.2. Microstructural Analysis

**Figure 6:**
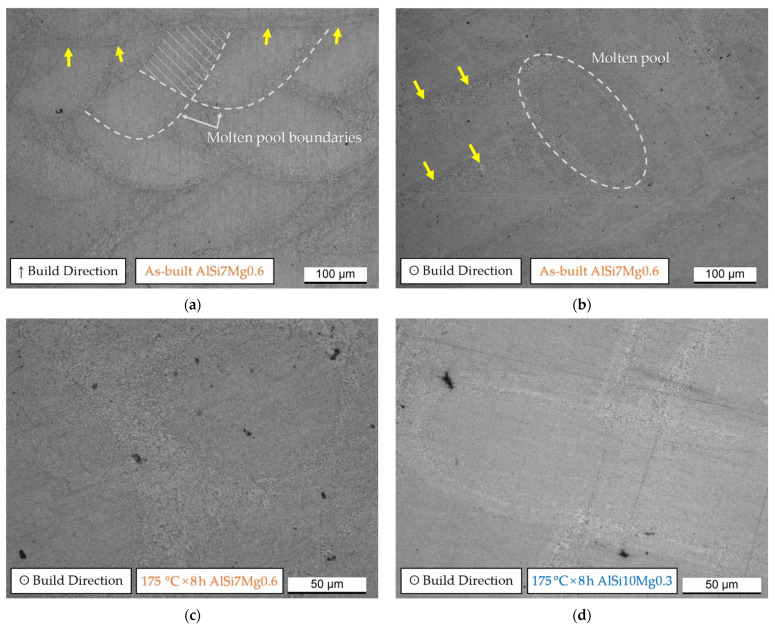
OM micrographs showing the as-built AlSi7Mg0.6 microstructure on the xz (**a**) and xy (**b**) planes, namely on the planes parallel (↑ Build Direction) and perpendicular (**ʘ** Build Direction) to the build direction, respectively. (**c**,**d**) OM micrographs of the AlSi7Mg0.6 (**c**) and AlSi10Mg0.3 (**d**) after the direct aging at 175 °C × 8 h (**ʘ** Build Direction). The yellow arrows indicate the boundaries of the laser scan tracks.

The OM micrographs shown in Figure 6a clearly show the typical “fish scale” structure which is formed by the cross-section of the laser scans. Due to the scanning strategy (Figure 1) used during the L-PBF, this fish-scale is longitudinally interrupted by a laser scan track (indicated in Figure 6a by yellow arrows). The same laser scan tracks are observable on the xy plane (Figure 6b), as well as the molten pool characterized by the typical ellipsoidal shape which is highlighted through the dotted ellipse. Figure 6c,d compares the microstructures of the AlSi7Mg0.6 and AlSi10Mg0.3 samples heat-treated at 175 °C × 8 h. No differences can be observed at the considered magnification. In addition, the SL (Figure 6a) and DL (Figure 6c) samples do not show any microstructural differences as discussed in detail in [22] for the AlSi10Mg0.3 samples in as-built conditions. For this reason, only the SL samples will be considered in the following parts related to the discussion of the heat-treated microstructures. At the same time, the same conclusions will be obviously drawn after the discussion of the HV values in as-built conditions. 

**Figure 7:**
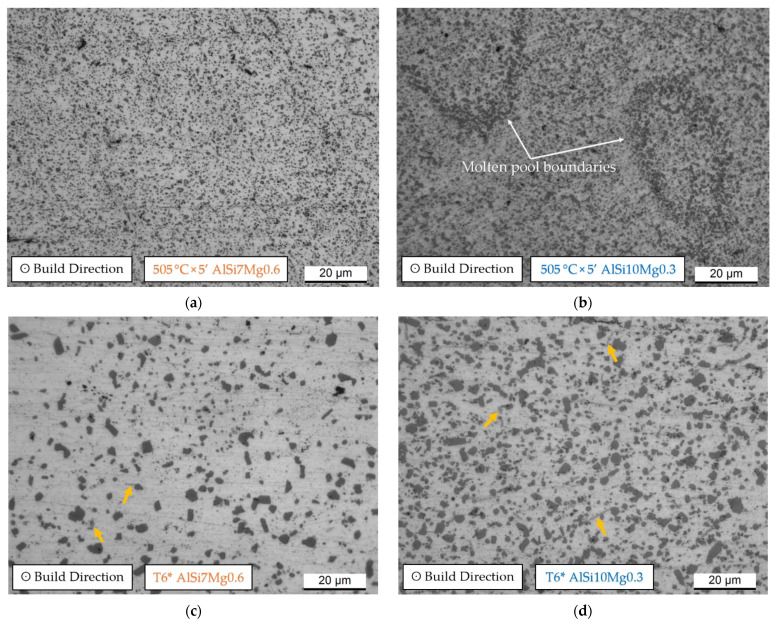
OM micrographs of the AlSi7Mg0.6 (**a**,**c**) and AlSi10Mg0.3 (**b**,**d**) microstructure after both the SHT at 505 °C × 5′ (**a**,**b**) and T6* (**c**,**d**). Orange arrows indicate different precipitates.

The microstructure obtained after the SHT (505 °C × 5′) is clearly affected by coarsening of the Si-eutectic particles (Figure 7a,b) and it became more homogenized only after 505 °C × 4 h (Figure 7c,d). As a matter of fact, the molten pool boundaries exhibited by the AlSi10Mg0.3 heat-treated samples for 5′ (Figure 7b) disappear after 4 h (Figure 7d). In contrast, the solution heat-treated AlSi7Mg0.6 samples do not frequently exhibit structures related to the molten pool or laser scan tracks on the xy and/or xz planes already after 5′. On the other hand, both aluminium alloys show clearly different precipitates which are indicated by orange arrows in Figure 7c,d. Further microstructural investigation should be performed in future works. At the same time, the precipitate having an elongated morphology can be associated with the brittle iron intermetallics, while the more spherical precipitate present within the AlSi7Mg0.6 α-Al matrix can be classified as Fe-Si-Mg intermetallics (Figure 6c) [16,29,30]. 

**Figure 8:**
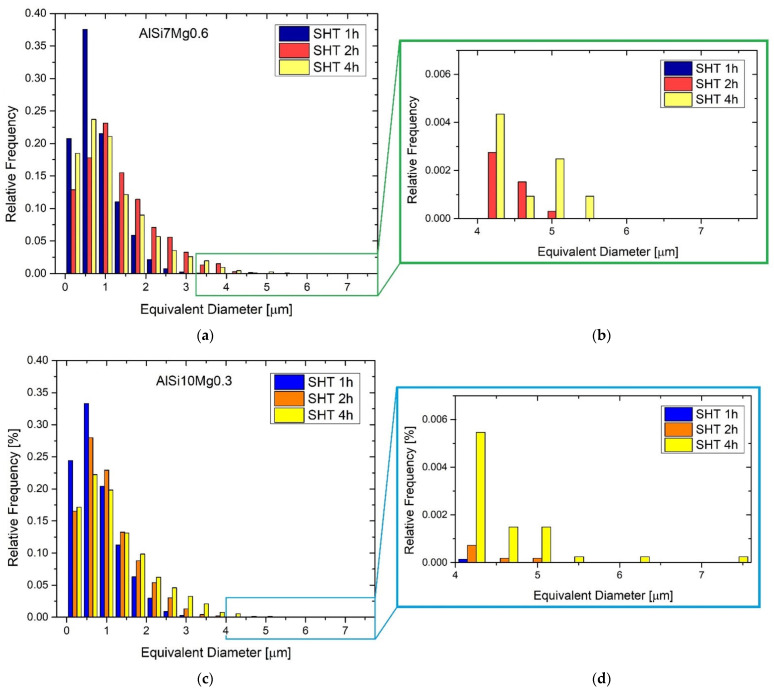
Statistical analysis of the equivalent diameter of the Si-eutectic particles analysed on: (**a**,**b**) AlSi7Mg0.6 and (**c**,**d**) AlSi10Mg0.3 samples solution heat treated at 505 °C × 1 h, 2 h and 4 h. The graphs in panels (**b**,**d**) are the magnifications of the same graphs in (**a**,**c**).

Si-eutectic particles were analysed in both the AlSi7Mg0.6 (Figure 8a,b) and AlSi10Mg0.3 (Figure 8c,d) sample solutions, heat-treated at 505 °C × 1 h, × 2 h and × 4 h, respectively. The statistical analysis related to the AlSi7Mg0.6 (Figure 8a) shows a displacement of the equivalent diameters, ranging from 0.2–3.4 μm (SHT 1h) to 0.2–5 μm and 5.4 μm for the SHT 2h and SHT 4h, respectively. At the same time, the Si particles per unit of area decrease from 0.076 to 0.043 (μm^−2^) while the average equivalent diameter increases from 0.81 to 1.32 μm. Despite the difference in holding time of 2 h at 505 °C between the red (SHT 2h, Figure 7b) and yellow (SHT 4h, Figure 8b) trends remain almost comparable except for the SHT 4h samples which show higher frequencies of Si-eutectic particles having equivalent diameters between 4.2 and 5.4 μm. As a matter of fact, SHT 4h confers higher coarsening phenomena of Si-eutectic particles. The same trends are described by the AlSi10Mg0.3 (Figure 8c,d) after the same HT conditions. In detail, the equivalent diameter range increases from 0.2–4 μm (SHT 1h) to 0.2–5 μm and 7.4 μm for SHT 2h and 4h, respectively. The Si particles per unit of area decrease simultaneously from 0.092 to 0.054 (μm^−2^) while the average equivalent diameter increases from 0.82 to 1.23 μm. Comparing Figure 8d,b, the AlSi10Mg0.3 samples heat-treated at 505 °C × 2 h and 4 h show the same trends illustrated by the AlSi7Mg0.6.

### 3.3. Microhardness Profiles in As-Built and Direct Aged Conditions

**Figure 9:**
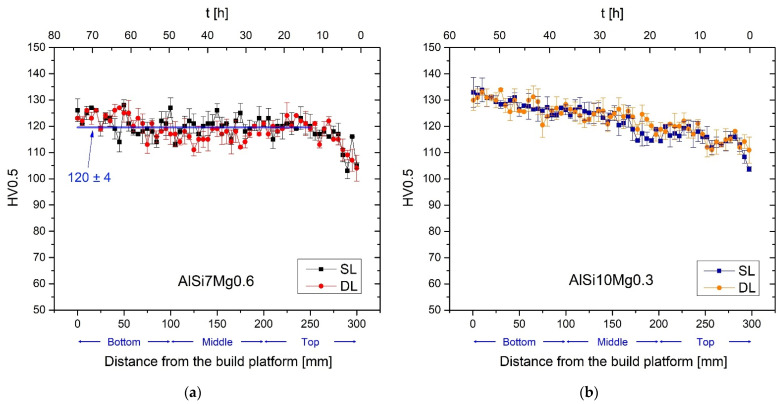
HV profiles from bottom to top regions of the AlSi7Mg0.6 (**a**) and AlSi10Mg0.3 (**b**) SL and DL bars.

Figure 9a illustrates the HV profiles of the SL and DL AlSi7Mg0.6 bars in as-built conditions. Both profiles were performed along the build direction (z-axis) and highlight the perfect overlap between the trends related to the SL and DL bars. As a matter of fact, the bottom and middle regions are characterized by HV values remaining constant at around (120 ± 4) HV0.5 for both the SL and DL bars. Only considering the farthest region of the same bars from the build platform, namely the last 200–300 mm, do the HV values decrease. AlSi10Mg0.3 SL and DL bars (Figure 9b) show the same decreasing trends, which are, however, extended from the bottom to the top regions. In this case, the HV measurements decrease from (133 ± 1) HV0.5 and (131 ± 2) HV0.5 to (108 ± 4) HV0.5 and (112 ± 3) HV0.5 for the SL and DL cases, respectively. Because both the SL and DL trends (Figure 9) perfectly overlap, only the SL samples will be considered in the following part related to the heat-treatments, as also reported in Section 3.2. Finally, the top regions of the AlSi7Mg0.6 and AlSi10Mg0.3 HV profiles, respectively, can be perfectly overlapped. Considering the as-built top and bottom samples, the anisotropy in HV measurements between the xy (Table 5) and xz (Figure 9a) planes are not detectable.

**Figure 10:**
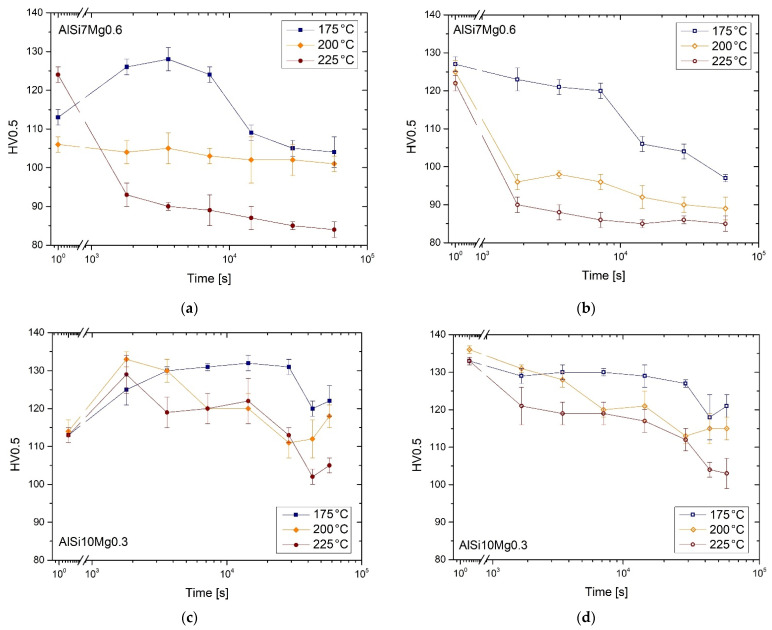
Aging curves of the top (**a**,**c**) and bottom (**b**,**d**) samples of the AlSi7Mg0.6 (**a**,**b**) and AlSi10Mg0.3 (**c**,**d**) alloys direct aged at 175 °C, 200 °C and 225 °C. The as-built conditions are considered at time equal to 1, and not to 0, because the time-axes are in log scale.

All aging curves related to both AlSi7Mg0.6 (Figure 10a,b) and AlSi10Mg0.3 (Figure 10c,d) are illustrated in Figure 10, where the filled and empty symbols are related to the top (Figure 10a,c) and bottom (Figure 10b,d) samples, respectively. Focusing on the top AlSi7Mg0.6 samples, only those with DA at 175 °C exhibit an increment in HV values up to the peak aging at a holding time of 2 h. After this, the HV measurements tend to decrease down to (104 ± 4) HV0.5, values less than (113 ± 2) HV0.5 of the as-built top sample. On the other side, the top sample DA at 225 °C immediately exhibits a fast decrease from (124 ± 2) HV0.5 to (84 ± 2) HV0.5 after 16 h because the considered top sample was 240–260 mm from the hot build platform, unlike the top samples considered for the DA at 175 °C (260–280 mm) and 200 °C (280–300 mm). In the latter case, the DA does not induce any variation in terms of HV measurements except for a slight decrease in HV values from (106 ± 2) HV0.5 (as-built) to (101 ± 2) HV0.5 (200 °C × 16 h). Considering the aging profiles (Figure 10b) related to the bottom samples, no peak-aging can be observed. As a matter of fact, the as-built HV values decrease of about 24%, 29% and 30% after the DA at 175 °C, 200 °C and 225 °C × 16 h, respectively. 

Top AlSi10Mg0.3 samples (Figure 10c) DA at 175 °C, 200 °C and 225 °C × 0.5–16 h, respectively, exhibit different aging responses from the AlSi7Mg0.6 due to the different as-built HV values at the same distance from the hot build platform (Figure 9). The DA at 175 °C × 4 h confers a peak aging at (132 ± 2) HV0.5, where the DA at 200 and 225 °C induces the peak aging conditions already after 0.5 h reaching (133 ± 2) HV0.5 and (129 ± 5) HV0.5, respectively. These results are consistent with [12,31]. Furthermore, in this case, the bottom AlSi10Mg0.3 samples show a decreasing trend for all DA temperatures considered. Table 5 summarize the HV microhardness values, and their variations, in as-built, peak-aging conditions, and after 16 h of HT.

### 3.4. Microhardness Profiles after Solubilization and T6 Heat-Treatments

**Figure 11:**
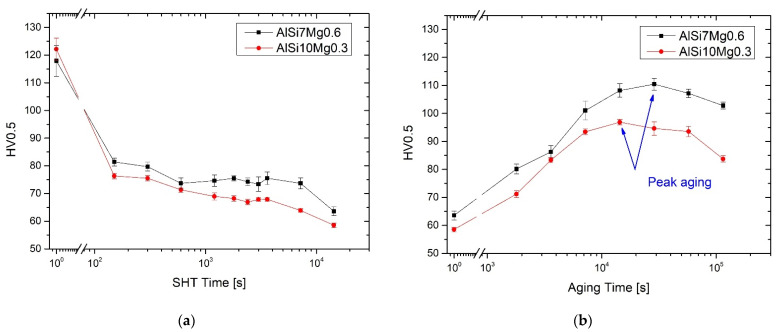
HV measurements on AlSi7Mg0.6 and AlSi10Mg0.3 samples after the SHT at 505 °C × 2.5′–4 h (**a**) and after the artificial aging at 175 °C × 0–32 h (**b**). The as-built conditions are considered at time equal to 1, and not to 0, because the time-axes are in log scale.

Figure 11a compares the HV profiles of the AlSi7Mg0.6 (black line-symbols) and AlSi10Mg0.3 (red line-symbols) alloys solution heat treated at 505 °C for 2.5′–4 h. Already starting from 2.5′ of treatment time, the HV values fall from (118 ± 6) HV0.5 and (122 ± 4) HV0.5 to (81 ± 1) HV0.5 and (76 ± 1) HV0.5 for AlSi7Mg0.6 and AlSi10Mg0.3, respectively. Increasing the SHT time up to 4 h, the continuous decreasing trends of the HV values reach (62 ± 2) HV0.5 and (59 ± 1) HV0.5 in the former and latter cases, respectively. In addition, both profiles remain basically parallel to each with a range of (5.8 ± 2.6) HV0.5. 

After the AA at 175 °C, the maximum reductions after the SHT 4h are partially recovered in 4 h and 8 h for AlSi10Mg0.3 and AlSi7Mg0.6 alloys as illustrated in Figure 10b. In the former case, the peak aging reaches (97 ± 1) HV0.5, which is always below the as-built microhardness values reported in Figure 9b. In the latter one, the AA at 175 °C × 8 h confers a microhardness of about (111 ± 2) HV0.5 that is perfectly confrontable to the top AlSi7Mg0.6 samples in as-built conditions (Figure 9a). Rao et al. [12] reported 64 HV and 58 HV after a SHT at 535 °C × 1 h and × 8 h, respectively. Both microhardness values are recovered after AA HTs at 165 °C × 6 h reaching the peak-aging at 120 HV, and at 180 °C × 2 h with 118 HV of peak-aging. After the same SHT at 535 °C × 1 h, Casati et al. [31] illustrated 78 HV increasing at 113 HV after an AA at 160 °C × 4 h. Higher HV values are discussed by Vanzetti et al. [20], who studied an SHT at 540 °C × 15′ (102 ± 5 HV) followed by an AA at 170 °C × 6 h (132 ± 3 HV). 

### 3.5. Mechanical Properties

**Figure 12:**
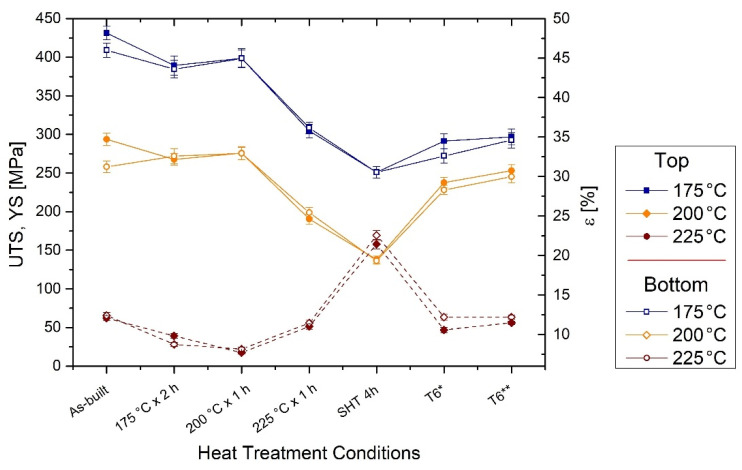
UTS, YS and elongation (ε) values of the AlSi7Mg0.6 samples in as-built conditions, and after the optimal conditions of the DA, solution, T6* (SHT + 175 °C × 4 h) and T6** (SHT + 175 °C × 8 h) heat treatments.

To complete the mechanical characterization of the AlSi7Mg0.6 samples heat treated at optimal conditions, Figure 12 shows the obtained UTS, YS and elongation values from the tensile testing at room temperature. 

Focusing on the as-built values, the HV variation between the bottom (full symbols) and top (empty symbols) samples is again confirmed by both the UTS and YS values. In fact, the bottom samples are characterized by a UTS of (431 ± 9) MPa and YS of (294 ± 8) MPa unlike the (409 ± 9) MPa and (258 ± 8) MPa shown by the top samples. As highlighted by the HV measurements performed on the DA samples, also the DA at 175 °C × 2 h, 200 °C × 1 h and 225 °C × 1 h homogenize the tensile strengths between the top and bottom samples. In addition, if the first and second HT conditions confer a maximum decrease of −9.5% and −8.8% of UTS and YS for the bottom samples, respectively; the third condition reduces them to about −29.5% and −35.4%. Table 6 summarizes the other variations also in terms of elongations which follow the inverse trends of the UTS and YS. 

The highest detrimental effects were conferred by the SHT (505 °C × 4 h) because the UTS and YS reach (251 ± 7) MPa and (138 ± 4) MPa, respectively. On the other hand, the elongation values exponentially increase up to 21.4 ± 0.6% and 22.5 ± 0.6% for the bottom (+77.7%) and top (+81.6%) samples, respectively. Focusing on the T6* (SHT + 175 °C × 4 h), the aging phenomena increase the UTS and YS at maximum values of (291 ± 10) MPa and (238 ± 7) MPa, respectively. The UTS reaches the YS of the as-built values, while the T6* YS shows an increment of + 100 MPa with respect to the SHT samples. On the other hand, the AlSi10Mg0.3 in the peak-aging conditions (T6*) (Figure 11b) shows the same UTS (288 ± 7 MPa) and YS (234 ± 7 MPa) values, as reported and discussed in [18]. As regards the AlSi7Mg0.6 samples in peak-aging conditions (T6**), the UTS and YS reach the maximum values of (297 ± 10) MPa and (253 ± 8) MPa, respectively. At the same time, in all considered cases, the AA confers detrimental effects on the elongations shown by the SHT samples. In fact, the ductility reaches the same as-built values. 

## 4. Discussion

### 4.1. Density Analysis

Both AlSi7Mg0.6 and AlSi10Mg0.3 samples in as-built conditions are characterized by high values of density (>2.676 g/cm^3^), namely by a restrained vol.% of pores which correspond to a $\rho_{r}$ higher than 99.83%. For these reasons, the process parameters (Table 2) also used to manufacture the AlSi7Mg0.6 samples can be considered as optimized values to obtain high-quality samples. The results are consistent with those reported in [9] for the AlSi10Mg0.3 and in [21,32,33] for AlSi7Mg0.6. While increasing the HT temperatures, the density values tend to decrease due to the synergetic effects between the increase in the number of total pores (vol. %) (Figure 5) and the growth of the area of pre-exiting pores formed during the L-PBF process. The analysed pores in as-built AlSi7Mg0.6 bar are classifiable as metallurgical pores (equivalent diameter < 100 μm), namely as pores formed by the gas enclosed within the molten pool during the LPBF process. Therefore, at high temperatures reached by an SHT, the material around a pore can be deformed due to the following synergetic effects: increase in the internal pressure and decrease in the yield strength of the material around the pore [25,34,35]. These effects are the same reported and discussed in [25] for the AlSi10Mg0.3 alloy. Therefore, the same research article can exactly explain the formation of pores during the AM process, and its variation during the HT in AlSi7Mg0.6 samples. In addition, the build platform heated to 150 °C already influences the dimensions of the pores as reported in (Figure 5a). The bottom region is characterized by a lower number of pores, characterized by higher dimensions, than the top regions, as also discussed in as-built AlSi10Mg0.3 bars in [25]. All of this, however, does not reflect a density variation between the bottom and top samples because the pores dimension compensates for their number, and vice versa. 

Finally, all density values obtained for the AlSi7Mg0.6 do not influence the HV measurements thanks to the maximum Δ$\rho_{r}$ = 0.05 g/cm^3^. In fact, only a Δ$\rho_{r}$ > 0.3 g/cm^3^ can influence the indentations and the correlated HV values due to the high distribution of pores within the material, as carefully demonstrated in [25]. The presence of metallurgical and LOF pores generates worsening effects on the ductility values and on the fatigue life [36,37] will be discussed in Section 4.3. 

### 4.2. Effects of Direct Aging Heat Treatments

The as-built AlSi7Mg0.6 and AlSi10Mg0.3 bars show comparable HV profiles that highlight the precipitation phenomenon induced by the build platform heated at 150 °C, as discussed in [22]. In fact, the AlSi10Mg0.3 exhibits a continuous decreasing trend, where the HV values vary from (132 ± 4) HV0.5 to (110 ± 5) HV0.5, thanks to the precipitation phenomena related to both the β-Mg_2_Si phase and nano Si particles as also discussed in [22,25]. The same precipitation phenomena were shown in [9]. Di Egidio [19] and Yang et al. [35] showed the β-Mg_2_Si phase and Si particles already in AlSi7Mg0.6 samples direct aged at 160 °C × 4 h and 8 h, respectively. 

**Figure 13:**
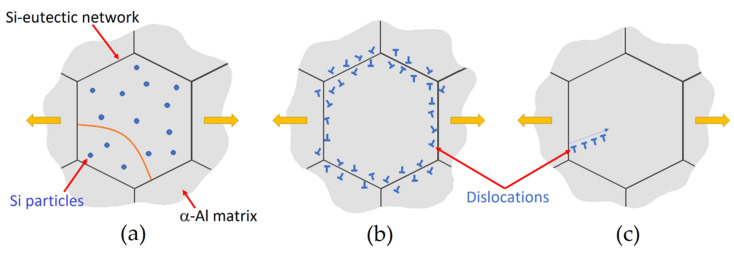
Graphical representation of the interaction between the dislocations and: (**a**) the Si particles within the α-Al matrix, and (**b**,**c**) the Si-eutectic network (Reprinted from reference [9]).

Focusing on the as-built AlSi7Mg0.6 bars, the bottom region could be characterized by an over-aging phenomenon, unlike the top where the decreasing trend of the as-built HV values (Figure 9a) can confirm a lower precipitation phenomenon. This is also confirmed by the aging curves related to the bottom and top samples where the former shows a constantly decreasing trend (Figure 10b,d), while the latter shows peak aging conditions (Figure 10a,c). The study on the effects induced by the hot build platform on the AlSi10Mg0.3 bar supports these considerations [22]. Finally, the increasing trend of the YS shown by the top AlSi7Mg0.6 (see Figure 12 and Table 5) directly aged at 175 °C × 2 h (+5.3%) and 200 °C × 1 h (+6.7%) which can confirm the precipitation effects. Considering the following sum [9]:

(4) $$YS=\sigma_{f}+\sigma_{SS}+\sigma_{HP}+\sigma_{Or}+\sigma_{\rho }+\sigma_{Pre}$$

where the $\sigma_{f}$ [MPa] is the friction stress of the lattice, $\sigma_{SS}$ [MPa] is the SSS strengthening, $\sigma_{HP}$ [MPa] is the strength obtained by the grain size (Hall–Petch equation), $\sigma_{Or}$ [MPa] is the Orowan strengthening, $\sigma_{\rho }$ is the dislocation hardening and $\sigma_{Pre}$ is the contribution conferred by the precipitates and dislocations, the strengthening effects which contribute to *YS* improvement are attributable to the precipitation phenomenon. In relation to the first and second strengthening terms, DA cannot increase their contribution due to the reduction of the solid solution and the consequent decrease of the friction stress [38]. The reduction of Mg and Si chemical concentrations $\left(C_{\alpha -\mathrm{Al}}^{\mathrm{Mg}};C_{\alpha -\mathrm{Al}}^{\mathrm{Si}}\right)$ in the α-Al matrix promotes the reduction of the $\sigma_{SS}$, as described by the following equation:

(5) $$\sigma_{SS}=k_{\mathrm{Mg}}{\left(C_{\alpha -\mathrm{Al}}^{\mathrm{Mg}}\right)}^{m}+k_{\mathrm{Si}}{\left(C_{\alpha -\mathrm{Al}}^{\mathrm{Si}}\right)}^{m}$$

where k and m are two constants related to Mg and Si alloying elements [9]. The decreasing in chemical concentration promotes a decrease in material strengthening [39]. In relation to the Hall–Petch equation, the low temperatures reach during the DA do not induce grain coarsening effects and, consequently, a strengthening variation [40]. The main causes of the YS improvement are related to the following sum:

(6) $$\sigma_{Or}+\sigma_{\rho }+\sigma_{Pre}$$

where their enhancement is conferred by the increase of the precipitate volume fraction $\left(\sigma_{Pre}\right)$ and their interactions with the dislocation movement $\left(\sigma_{Or}\right)$ as widely described in [9]. In relation to the $\sigma_{Pre}$ contribution, its following mathematical model combines the volume fraction (f) of the precipitates and their dimensions $\left(l_{D},\;l_{t}\right)$, as follows:

(7) $$\sigma_{Pre}=C\frac{Gb}{{\left(l_{D}l_{t}\right)}^{1/2}}\left[f^{1/2}+0.70{\left(\frac{l_{D}}{l_{t}}\right)}^{1/2}+0.12\left(\frac{l_{D}}{l_{t}}\right)f^{3/2}\right]$$

where C [-] is a material constant, G [GPa] is the shear modulus of Al (G=25.4 Gpa, [41]), b is the Burger vector (b=0.286 nm, [9]), $l_{D}$ and $l_{t}$ are the diameter and thickness of the precipitates, respectively. Equation (7) highlights an increase in material strengthening when the volume fraction (f) increases, as is possible to detect in peak-aged samples [9,20,22,26,36]. The same results can be also obtained modifying the $l_{D}$ and $l_{t}$ values. With the number of obstacles consequently increasing, the dislocation–precipitate interactions rise during the plastic deformation. As a matter of fact, dislocations interact with the Si particles dispersed within the α-Al matrix (Figure 13a), and with the Si-eutectic network (Figure 13b), which can promote the emission of other dislocations (Figure 13c) [9]. Considering that the Si-eutectic network breaks in Al-Si-Mg samples DA at temperatures higher than 175 °C, the only increase in tensile strength and HV microhardness, with respect to the as-built conditions, can be conferred by the precipitation phenomena. In this scenario, the damaging effects of the Si network previously discussed for the DA at 200 °C, and the precipitation phenomena, can confirm the constant trend of the Vickers measurements (Figure 10a). The extensive damaging effects of the Si network and the coarsening phenomena conferred by the DA at 225 °C cannot promote an increase in Vickers microhardness and tensile strengths. 

In this scenario, the Orowan mechanism explains the material strengthening where the interactions between a dislocation line and different impenetrable precipitates promote the formation of dislocation loops around the same particles, consequently increasing the strengthening of the material [9,42]. Considering the Si-precipitates, the Orowan contribution in Al-Si alloys can be estimated as proposed by [43], as follows:

(8) $$\sigma_{Or}^{\mathrm{Si}}=\frac{\varphi Gb}{d_{\mathrm{Si}}}{\left(\frac{6f_{\mathrm{Si}}}{\pi }\right)}^{\frac{1}{3}}$$

where φ [-] is a material constant, G [GPa] is a shear modulus, b is a Burger vector, $d_{\mathrm{Si}}$ is the diameter of Si-particle and $f_{\mathrm{Si}}$ is the volume fraction of the Si particles. Hadadzadeh et al. [44] affirmed that the β-Mg_2_Si precipitates act a material strengthening similar to the Si precipitates that increases the results obtained through Equation (4) of about ~13 MPa. Li et al. [45] obtained an increment of (27 ± 2) MPa. Because the β-Mg_2_Si precipitates follow the Orowan looping, the obtained material strengthening contributions are given by:

(9) $$\sigma_{Or}^{{\mathrm{Mg}}_{2}\mathrm{Si}}=M\frac{0.4Gb}{\sqrt{1-\vartheta }}\frac{\ln\left(2\sqrt{\frac{2}{3}}R\right)}{\lambda }\;b{\left(\frac{R}{b}\right)}^{\left(\frac{3m}{2}-1\right)}$$

where M is the Taylor factor (*M* = 3.06, [9,45]), b is the Burger vector, G [GPa] is the shear modulus, ϑ is the lattice parameter mismatch at room temperature, λ is the distance between two adjacent particles, R is the average precipitate diameter and m is the Poisson’s ratio of aluminium (m = 0.34–0.35, [45,46]). This confirms our hypothesis on the precipitation effects correlated to the variation of the YS in Equation (4). Lastly, the term $\left(\sigma_{\rho }\right)$ related to the dislocation hardening (i.e., to the dislocation density [47]) is subjected to the following contrasting effects: the relief of the internal residual stresses with the decrease in dislocation number, and its increase due to the dislocation motion and pile up around the precipitates [9,43,48,49,50]. 

A further microstructural investigation will be surely performed in future works to better characterize the microstructure before and after the HTs, and to detail their effects in relation to the discussion reported above. 

Several DA HTs were performed to homogenize the differences between the bottom and top regions (Figure 9 and Figure 12) while preserving the high mechanical performance conferred by the LPBF process. Already after the DA at 175 °C × 2 h, the HV measurements for top and bottom AlSi7Mg0.6 samples are homogenized thanks to the intersection of both the over-aging conditions. In fact, if the bottom sample shows an over-aging phenomenon thanks to the absence of a peak-aging (Figure 10b), the top sample reaches it only after the peak-aging at 1 h. Focusing on the DA at 200 °C and 225 °C, 1 h of treatment can represent the optimal HT conditions for HV homogenization. In addition, the tensile test performed on this optimal HT conditions confirm the homogenization in terms of UTS, YS and elongation (Figure 12). At the same time, the first and second DA do not confer detrimental effects on the as-built properties unlike the DA at 225 °C × 1 h where the coarsening phenomena of the Si-eutectic network occurs [22]. In a reduced way, the latter effect can be also characterized by the samples direct aged at 200 °C because the top samples do not show significant variations in terms of HV measurements. Therefore, the damaged Si-eutectic network can balance the hardening effects of the precipitation phenomena. Similar conditions were reported by the bottom and top AlSi10Mg0.3 samples which homogenized their HV values after 1 h of holding time at 175 °C, 200 °C and 225 °C. 

### 4.3. Effects of Solubilization and T6 Heat Treatments

**Figure 14:**
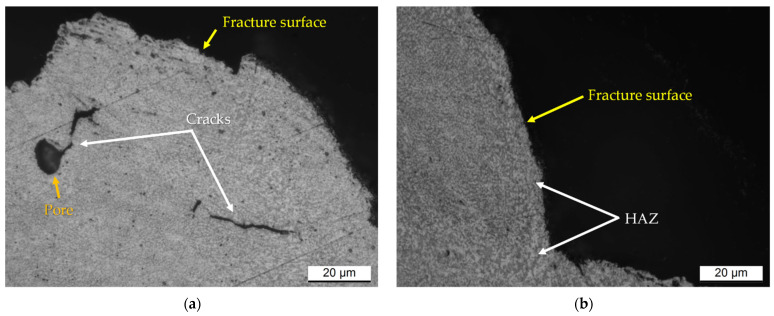
Longitudinal sections of the as-built AlSi10Mg0.3 samples showing: (**a**) the crack initiation from a pore, (**b**) the crack propagation along the HAZ.

SHT deletes the fine as-built microstructure of both the AlSi7Mg0.6 and AlSi10Mg0.3 subjected to LBPF (Figure 6 and Figure 7) already after 2.5′ of treatment. Due to the high reached temperature (505 °C), initially, the Si-eutectic network begins to show different openings where the α-Al matrix interconnects two adjacent cells as shown in Figure 14b. Then, the Si particles grow on the remaining Si-eutectic network until its complete destruction [9]. As the SHT time increases, the growth of the globular Si-eutectic particles proceeds (Figure 6) and, consequently, the Si-particles per unit area decrease. As a matter of fact, the maximum equivalent diameters increase from 3 μm (SHT 1h) to 5.4 and 7.4 μm for the SHT 4h AlSi7Mg0.6 and AlSi10Mg0.3 alloys, respectively. Joining together this coarsening phenomenon to the recrystallization process of the nano-size grains of the as-built samples [40,49], the HV measurements are designed to decrease (Figure 11a). The same decreasing trend is exhibited by the tensile samples, where the UTS and YS decreased by −48.8% and −53.1%, respectively. 

In addition to these detrimental effects, the SHT also promotes an increment of pore size and a formation of new pores as previously discussed and reported by [23,24]. In other words, it promotes a decrease in density from 2.675–2.677 g/cm^3^, which defines the as-built samples as high-quality or fully-dense samples, to 2.663 g/cm^3^ and 2.651 g/cm^3^ for AlSi7Mg0.6 and AlSi10Mg0.3 samples, respectively. Despite these reached densities, the HV measurements were not influenced as discussed in Section 4.1. On the other hand, the presence of defects influences the ductility of both the as-built and heat-treated AlSi7Mg0.6 and AlSi10Mg0.3 samples [36,37,48]. Metallurgical and LOF pores were often revealed within both the fractured MP boundaries and laser scan segments of the as-built AlSi10Mg0.3 samples, as analysed by [48], and they can be consequently considered as trigger points of the cracks. Figure 14a shows the same findings. After the crack initiation, it generally propagates and grew nearly the MP and/or laser scan tracks boundaries where the HAZ (Heat-Affected Zone) offers a less resistant Si-eutectic network (Figure 14b). In this context, the occurrence of fracture in the laser scan tracks was also observed in AlSi10Mg0.3 horizontal samples by [48]. Through SEM investigations, [48] also demonstrated that the crack path extends along the α-Al cell boundaries where the Si-particles are easily connected. From a three-dimensional point of view, these crack paths lead to the layer-layer or intra-layer fracture [9] The analogous fracture mechanism described the direct aged AlSi7Mg0.6 and AlSi10Mg0.3 samples thanks to their microstructural morphology comparable with the as-built samples [18,19,26,51]. 

**Figure 15:**
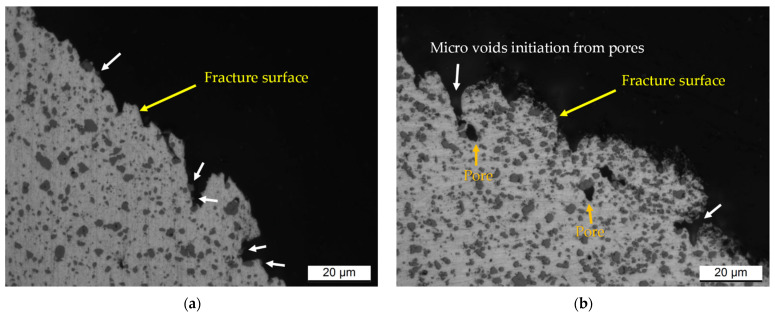
Longitudinal sections of the T6** AlSi10Mg0.3 samples showing: (**a**) the Si-particles attached (white arrows) at the fracture surface, (**b**) the pores and the micro-voids initiation from pores.

Focusing on the T6 heat-treated samples, the cracks principally interconnect the coarse Si-particles due to their decohesion phenomena from the α-Al matrix. For these reasons, the Si-eutectic particles (indicated by white arrows in Figure 15a) remain often attached at fracture surface. At the same time, the ductile α-Al matrix seems to hinder the crack propagation. The same findings are shown by [19,48]. Furthermore, in T6 heat-treated Al-Si-Mg samples, the pores can promote the crack initiation as highlighted by [19], who exhibited the same fracture profile and structure shown in Figure 15b. In addition, the presence of MP and/or laser scan tracks boundaries (Figure 7b), remain even after the SHT, control the crack propagation as widely discussed in [18], where the T6** heat-treated AlSi10Mg0.3 samples were analysed. In fact, the crack can easily interconnect the micro voids formed by the decohesion phenomena where the Si-eutectic particles are very close to each other. The MP or laser scan track boundaries satisfy this condition. 

The AA at 175 °C recovers the HV measurements after the SHT 4h thanks to the precipitation phenomena correlated to the β-Mg_2_Si. The T6** AlSi7Mg0.6 samples reach (111 ± 2) HV0.5, values that characterize the peak-aging (Figure 10b) and the top bar in as-built conditions (Figure 9). On the other hand, the peak-aging shown by the T6* AlSi10Mg0.3 samples do not reach again the microhardness of the top as-built samples with the (97 ± 1) HV0.5. Despite the differences in terms of the Si wt.%, the difference of about (14 ± 3) HV0.5 between both the peak-aging conditions is conferred by the different content of Mg which can promote several amounts of β-Mg_2_Si phase during the AA. Always in relation to the Mg content, the SHT AlSi7Mg0.6 samples (Figure 11) show higher HV values than AlSi10Mg0.3 alloy in the same conditions due to the higher Mg percentage in solid solution, as confirmed by [52] and through the Equation (4). 

In conclusion, although the improvement of HV values of the AlSi7Mg0.6 alloy in peak aging conditions, the UTS (~280 ± 5 MPa) and the YS (~230 ± 5 MPa) remain lower than the UTS and YS values of the as-built, DA at 175 °C × 2 h and DA at 200 °C × 1 h top and bottom samples. In addition, the T6* heat-treated samples do not show any enhancement in terms of ductility, contrary to the expectations highlighted in the Introduction. As a matter of fact, the elongation values are fully comparable to those obtained for the as-built and DA samples (Figure 12). 

**Figure 16:**
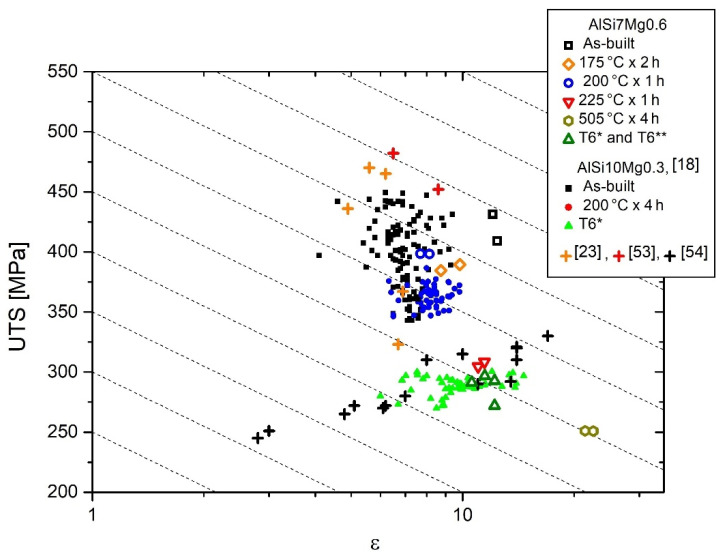
QI-graph where the elongation and the ultimate tensile strengths of as-built and heat-treated AlSi7Mg0.6 samples are compared to the results reported in [18]. (Adapted from reference [18]).

To obtain an effective evaluation of the optimal HT conditions, the *UTS* and elongation (*ε*) values can be combined defining the *QI* (Quality Index), as follows:

(10) QI=UTS+dlog(ε)

where d (equal to 150) is an empirical parameter that makes the *QI* independent of the YS. The results obtained were plotted in the same QI-graph reported in [18] for the AlSi10Mg0.3 alloy (Figure 16). Also in this case, the as-built AlSi7Mg0.6 samples are characterized by higher QI values (570–595 MPa) than the DA samples at 175 °C × 2 h (526–538 MPa) and at 200 °C × 1 h (531–535 MPa). At the same time, these values are also higher than those discussed in [18] for AlSi10Mg0.3 samples and reported by Paul et al. [53]. On the other hand, the detrimental effects of the DA at 225 °C are highlighted by QI values (461–467 MPa) which are comparable with those referred to as the T6* and T6** HTs (435–456 MPa). These last values can be matched with the QI values of the T6 AlSi10Mg0.3 samples produced by casting [23,53,54] and LPBF [18] techniques, respectively. 

## 5. Conclusions

The present research article aims to evaluate the effects induced by different DA and T6 heat treatments on AlSi7Mg0.6 and AlSi10Mg0.3 alloys manufactured via the Laser Powder-Bed Fusion process. In this scenario, the following conclusions can be drawn:

1. The as-built HV values of AlSi7Mg0.6 do not greatly differ from those shown by AlSi10Mg0.3. Both couples of profiles perfectly overlap from a 200 mm bar height despite the differences in Si and Mg content.
2. There are no substantial differences between the SL and DL AlSi7Mg0.6 samples both in terms of as-built microstructure and mechanical properties. The proximity to the hot build platform induces an increment of HV values in the bottom rather than in the top regions thanks to the precipitation phenomena of Si particles and β-Mg_2_Si phase in AlSi10Mg0.3 bars. The same HV profile was, however, obtained for AlSi7Mg0.6 bars. In addition, the bottom regions are characterized by a lower number of pores, but having larger dimensions, than the top regions.
3. The differences in the mechanical performance between the bottom and top AlSi7Mg0.6 samples are levelled out by the DA at 175 °C × 2 h, which does not influence the ultimate tensile and yield strengths such as the DA at 200 °C × 1 h. The same results are obtained for AlSi10Mg0.3 samples after 1 h of the same direct aging heat treatments.
4. The SHT certainly confers high elongation values to AlSi7Mg0.6 samples, thus satisfying the current standard specifications (ε >> 10%). The worsening effects on UTS and YS are recovered through the subsequent artificial aging, but the elongations return again at the direct aged values. For these reasons, and considering the QI values, the direct aging heat treatments at the peak aging conditions confer better mechanical performance.

## Figures and Tables

**Table 1:** Nominal chemical composition (wt.%) of AlSi7Mg0.6 and AlSi10Mg0.3 gas-atomized powders.

Al	Si	Mg	Fe	Cu	Mn	Zn	Ti	C	H	N	O
Bal.	7.04	0.59	0.06	<0.005	0.006	0.011	0.12	0.03	0.002	<0.002	0.03
	10	0.31	0.12	0.001	0.005	0.002	0.04	<0.005	0.002	<0.002	0.10

**Table 2:** Process parameters used to manufacture AlSi7Mg0.6 and AlSi10Mg0.3 bars and billets.

Alloys	Laser Power (P, [W])	Scan Speed (v, [mm/s])	Layer Thickness (t, [μm])	Hatch Spacing (h, [μm])	Energy Density (ED, [J/mm^3^])	Build Platform [°C]
Skin	330	600	90	70	87.3	150
Core	370	1400			42.0	

**Table 3:** Heat treatment performed on AlSi7Mg0.6 and AlSi10Mg0.3 samples.

HT	Temperature	Time	Samples
DA	175 °C	30 min, 1 h, 2 h, 4 h, 8 h, 16 h	Top (280–300 mm) and Bottom (0–20 mm)
	200 °C		Top (260–280 mm) and Bottom (20–40 mm)
	225 °C		Top (240–260 mm) and Bottom (40–60 mm)
SHT	505 °C	2.5 min, 5 min, 10 min, 20 min, 30 min, 40 min, 50 min, 1 h, 2 h, 4 h	Other (60–260 mm)
T6	505 °C	4 h	
	175 °C	30 min, 1 h, 2 h, 4 h, 8 h, 16 h, 32 h	

**Table 4:** Standard errors that are associated to each pore area analyzed [25].

Areas	Standard Errors
$A_{p}<1\;{\mu m}^{2}$	10%
$1\;{\mu m}^{2}\leq A_{p}<10\;{\mu m}^{2}$	7%
$10\;{\mu m}^{2}\leq A_{p}<30\;{\mu m}^{2}$	6%
$A_{p}\geq 30\;{\mu m}^{2}$	4%

**Table 5:** Vickers microhardness values, and their variation, measured on the as-built and direct aged AlSi7Mg0.6 and AlSi10Mg0.3 samples. For each DA at 175, 200 and 225 °C, the HV measured at the peak-aging and after 16 h of treatment were listed.

**HT Conditions**		**AlSi7Mg0.6**				**AlSi10Mg0.3**			
		**Top**		**Bottom**		**Top**		**Bottom**	
		HV0.5	Δ [%]	HV0.5	Δ [%]	HV0.5	Δ [%]	HV0.5	Δ [%]
175 °C	0 h	113 ± 2	-	127 ± 2	-	113 ± 2	-	133 ± 1	-
	Peak-Aging	128 ± 3	+13.4	-	-	132 ± 2	+16.8	-	-
	16 h	104 ± 4	−18.8 ^1^	97 ± 1	−23.6	122 ± 4	−7.58 ^1^	121 ± 3	−9.0
200 °C	0 h	106 ± 2		125 ± 3	-	114 ± 3	-	136 ± 1	-
	Peak-Aging	-	-	-	-	133 ± 2	+16.7	-	-
	16 h	101 ± 2	−4.7	89 ± 3	−32.0	118 ± 3	−11.3 ^1^	115 ± 3	−15.4
225 °C	0 h	124 ± 2	-	122 ± 2	-	113 ± 2	-	133 ± 1	–
	Peak-Aging	-	-	-	-	129 ± 5	+14.2	-	-
	16 h	84 ± 2	−32.3	85 ± 2	−30.3	105 ± 2	−18.6 ^1^	103 ± 4	−22.6

^1^ The HV variation (Δ [%]) is calculated between the Peak-Aging and the HV values after 16 h.

**Table 6:** Variation in the mechanical properties between the as-built and heat-treated conditions for AlSi7Mg0.6 samples.

Samples	HTs	ΔUTS	ΔYS	HTs	ΔUTS	ΔYS
Top	175 °C	−6.0%	+5.3%	SHT	−38.7%	−47.2%
Bottom		−9.7%	−8.8%		−41.8%	−53.1%
Top	200 °C	−2.6%	+6.7%	T6*	−33.5%	−11.6%
Bottom		−7.6%	−6.1%		−32.4%	−19.1%
Top	225 °C	−24.6%	−23.0%	T6**	−28.4%	−4.9%
Bottom		−29.4%	−35.2%		−31.2%	−13.8%

## Data Availability

Not applicable.
